# Examining the association between paramedic transport to the emergency department and hospital admission: a population-based cohort study

**DOI:** 10.1186/s12873-021-00507-2

**Published:** 2021-10-12

**Authors:** Ryan P. Strum, Fabrice I. Mowbray, Andrew Worster, Walter Tavares, Matthew S. Leyenaar, Rebecca H. Correia, Andrew P. Costa

**Affiliations:** 1 Department of Health Research Methods, Evidence and Impact, McMaster University, CRL B106, 1280 Main Street West, Hamilton, ON L8S 4L8 Canada; 2 Department of Medicine, Division of Emergency Medicine, McMaster University, Hamilton, Canada; 3 The Wilson Centre, University of Toronto, Toronto, Canada; 4 York Region Paramedic and Senior Services, Regional Municipality of York, Newmarket, Canada; 5 Department of Medicine, McMaster University, Hamilton, Canada

**Keywords:** Hospitalization, Hospital admission, Paramedic, Paramedicine, Ambulance, Emergency medical services, Emergency department

## Abstract

**Background:**

Increasing hospitalization rates present unique challenges to manage limited inpatient bed capacity and services. Transport by paramedics to the emergency department (ED) may influence hospital admission decisions independent of patient need/acuity, though this relationship has not been established. We examined whether mode of transportation to the ED was independently associated with hospital admission.

**Methods:**

We conducted a retrospective cohort study using the National Ambulatory Care Reporting System (NACRS) from April 1, 2015 to March 31, 2020 in Ontario, Canada. We included all adult patients (≥18 years) who received a triage score in the ED and presented via paramedic transport or self-referral (walk-in). Multivariable binary logistic regression was used to determine the association of mode of transportation between hospital admission, after adjusting for important patient and visit characteristics.

**Results:**

During the study period, 21,764,640 ED visits were eligible for study inclusion. Approximately one-fifth (18.5%) of all ED visits were transported by paramedics. All-cause hospital admission incidence was greater when transported by paramedics (35.0% vs. 7.5%) and with each decreasing Canadian Triage and Acuity Scale level. Paramedic transport was independently associated with hospital admission (*OR* = 3.76; 95%*CI* = 3.74–3.77), in addition to higher medical acuity, older age, male sex, greater than two comorbidities, treatment in an urban setting and discharge diagnoses specific to the circulatory or digestive systems.

**Conclusions:**

Transport by paramedics to an ED was independently associated with hospital admission as the disposition outcome, when compared against self-referred visits. Our findings highlight patient and visit characteristics associated with hospital admission, and can be used to inform proactive healthcare strategizing for in-patient bed management.

**Supplementary Information:**

The online version contains supplementary material available at 10.1186/s12873-021-00507-2.

## Background

Hospital admissions have increased steadily over the past 10 years in Ontario, Canada [1]. High hospital bed occupancy inhibits emergency department (ED) throughput and has a direct downstream impact on the quality and timeliness of emergency care provided [2]. Hospital inpatient units have become increasingly congested and overburdened alongside increased patient complexity and occupancy loads [3, 4]. Ontario’s number of in-patient hospital beds have remained relatively the same over the past decade; the lowest of all Canadian provinces when measured in beds per capita (1.4 per 1000) [5]. Ontario’s yearly admissions now account for nearly half of all hospital admissions in Canada, despite being 38.7% of the national population [1, 6]

Understanding the factors that influence disposition decision-making may be important to reduce admissions. Higher admission rates are associated with older age, higher triage acuity, male sex, and certain chronic diseases (end-stage renal disease, chronic renal disease, congestive heart failure) [7–12]. While medical acuity and clinical conditions are the primary predictors of admission, additional non-medical characteristics (i.e., inadequate access to primary care, socioeconomic status, family member influences) may also contribute [13, 14].

Paramedic transport to ED has been established as a potential factor that influences ED disposition decisions, though extant literature has limited generalizability given heterogeneity in the magnitude of association, patient characteristics (e.g., age, non-urgent visits) used to adjust the association, and different models of care found between individual hospitals and jurisdictions [8–10, 15, 16]. As demands for paramedic utilization steadily increase in Ontario, [17, 18] a population-based analysis of patient characteristic associations with hospital admission is warranted to improve the precision of this relationship, and provide region-specific data for health system planning which is currently lacking in Ontario.

Our objective was to determine if an association exists between the mode of ED transportation (ambulance versus self-referral) and hospital admission using population-based data in Ontario, Canada. We hypothesized that the mode of transportation would have greater odds of hospital admission, after controlling for relevant patient and hospital characteristics.

## Methods

### Study design

We conducted a population-based retrospective cohort study by analyzing administrative ED records from the National Ambulatory Care Reporting System (NACRS) database. The STROBE statement was followed for reporting of results (Additional file 1) [19].

### Population

All adult patients aged (≥18 years) triaged in an Ontario ED and arriving by either paramedic transport or self-referral were included. We excluded patients who were not triaged by hospital staff (registered but left prior to triage), as hospital admission is not possible in this cohort and represent a very small and distinct cohort of patients. Furthermore, patients were excluded if their mode of transportation included any air ambulance transportation. ED visits that did not result in a discharge or admission from the ED were excluded (i.e. dead on arrival, triaged but left prior to physician assessment), as these outcomes are not relevant to the studies objective. Data for this study represents a population-based view of ED use and paramedic transport. No sampling methods were required, all records meeting eligibility criteria were included to minimize bias.

### Data sources

Data were extracted from the NACRS dataset, housed in the Institute for Clinical Evaluative Sciences (IC/ES), on eligible patients who visited an ED in Ontario between April 1, 2015 to March 31, 2020. This timeframe represents the most recently available five-year period prior to the COVID-19 pandemic, when paramedic and hospital utilization may have changed [20]. NACRS is a hospital and community-based ambulatory care administrative database that collects all patient visit data at the time of service [21, 22]. IC/ES is a non-profit, independent corporation that supports the study of health service and population-wide outcomes in Ontario using administrative databases.

### Variables and measurement

All patient characteristics included in this study were measured and recorded at the time of ED registration and selected based on the prior literature, clinical judgement and data availability. Characterises included sex, age, access to primary care, triage acuity, comorbidities, primary diagnostic category, ED geographic location, ED visit outcome, and repeat ED visits within 30 days. Variables were collapsed into ordinal and nominal categories to facilitate model stability when data were non-continuous and truncated (i.e., < 5% per cell of cohort).

Patient age was originally extracted as a twenty-level categorical variable, due to personal health information privacy restrictions. Age was further collapsed into three categories to parallel major age progressions (18–39, 40–64, 65–105 years). Access to primary care was recorded at the time of visit as the identifying physician overseeing the majority of primary healthcare. Data were also collected on population density, and classified as urban or rural.

Triage acuity was assigned by the ED triage nurse following ED registration, not by a paramedic, using the Canadian Triage and Acuity Scale (CTAS). CTAS is an ordinal scale that ranges from one to five, with a score of one indicating the most emergent (resuscitation) and five the least urgent (non-urgent) [23]. Triage acuity was condensed into three categories, similar to prior ED studies in Canada, [24] as CTAS score one (0.8%) and five (4.9%) are relatively infrequent: scores of one and two were grouped as ‘emergent’, scores of three as ‘urgent’, and scores four and five grouped as ‘non-urgent’.

Main diagnoses were assigned by an attending ED physician and recorded using the International Statistical Classification of Diseases and Related Health Problems, 10th revision (ICD-10). Comorbidities were recorded as pre-existing diagnoses at time of ED visit and included hypertension, diabetes, chronic obstructive pulmonary disease, asthma, rheumatoid arthritis, congestive heart failure, bowel disease, and cancer.

### Statistical analysis

Descriptive statistics were reported using measures of frequency and proportions and stratified between modes of transportation (paramedic and self-referral). Multivariable binary logistic regression was used to calculate the association between the mode of transportation and hospital admission status, after adjusting for age, sex, triage acuity, comorbidity count, population density, repeat ED use, and the presence of system-specific disease condition. Results were reported as crude and adjusted odds ratios to show independent associations of each characteristic, alongside corresponding 95% confidence intervals (CI). Data were managed and analyzed in **R** software (v.3.6). Missing data was scant (< 1%) and handled using pairwise deletion.

## Results

**Fig. 1:**
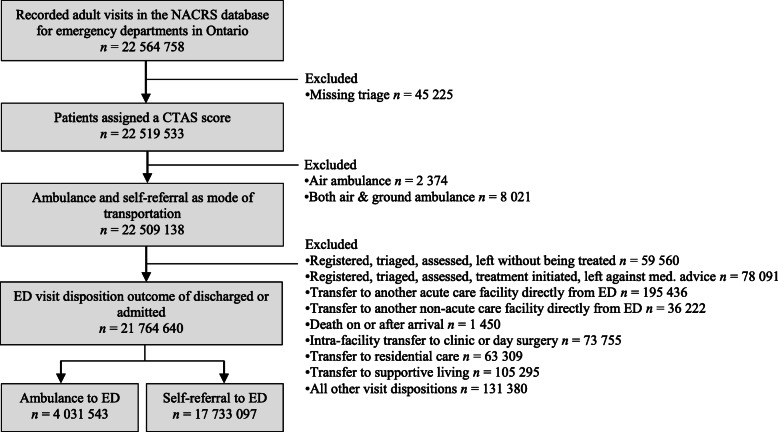
Flow diagram of patient inclusion for study of Ontarians assessed in the ED based on mode of transportation

Our cohort contained 21,764,640 adult patients presenting to an Ontario ED. The cohort yielded 4,031,543 (18.5%) patients transported by paramedics and 17,733,097 (81.5%) patients self-referred to ED. The flow of patients used to construct the cohort and reasons for exclusion are displayed in Fig. 1. Overall, 96.5% of the initial NACRS population was incorporated in this study’s cohort.

In this cohort, 12.6% (2,748,487) of all patients were admitted to the receiving hospital from ED, of which 51.4% (1,411,377) were transported by paramedics and 48.6% (1,337,110) by self-referral. The majority patients were: female, not assigned an emergent triage score, sought ED care at an urban facility, and did not return to the ED within 30 days. Notable patient characteristics differences were noted between the two modes of transportation in age group, acuity, diagnostic categorization, and visit outcome.

The majority of patients transported by paramedics were in the oldest age group (65 to 105 years, 53.3%). Non-emergent acuities constituted the smallest categorization of patients for paramedic transport (8.6%), while emergent was the smallest for self-referral (18.9%). Differences were noted between paramedic transport and self-referral in main diagnoses of mental and behavioural disorders (9.0, 3.7%), diseases of the circulatory system (7.7, 3.7%), diseases of the skin and subcutaneous tissue (1.1, 4.5%), and diseases of the musculoskeletal system and connective tissue (4.6, 8.1%). A comprehensive list of descriptive patient and visit characteristics are displayed in Table 1.

**Table 1:** Characteristics of patients assessed in the emergency department, by mode of transportation to emergency department, Ontario, Canada, April 1, 2015 to March 31, 2020

Characteristic	Paramedic Transport (n, %) ***n*** = 4,031,543	Self-referral (n, %) ***n*** = 17,733,097
**Sex**		
Male	1,865,952 (46.3)	8,145,480 (46.0)
Female	2,165,591 (53.7)	9,587,617 (54.0)
**Age**, years		
18–39	747,598 (18.5)	6,600,607 (37.2)
40–64	1,134,367 (28.1)	6,877,715 (38.8)
65–105	2,149,578 (53.3)	4,254,775 (24.0)
**Access to primary care**		
Family physician	3,485,471 (86.5)	15,423,577 (87.0)
Other physician classification	30,590 (0.8)	199,762 (1.1)
None	320,346 (7.9)	1,573,982 (8.9)
Unreported	195,136 (4.8)	535,776 (3.0)
**Triage Acuity**, CTAS		
Emergent [1, 2]	1,622,216 (40.2)	3,352,710 (18.9)
Urgent [3]	2,062,831 (51.2)	8,232,246 (46.4)
Non-urgent [4, 5]	346,496 (8.6)	6,148,141 (34.7)
**Comorbidities,** total^a^		
0–2	2,841,902 (70.5)	15,581,739 (87.9)
3–5	1,162,149 (28.8)	2,120,884 (12.0)
6–8	27,492 (0.7)	30,474 (0.1)
**Diagnostic Category,** ICD-10^b, c^		
A,B – Certain Infectious Diseases	132,987 (3.3)	590,440 (3.3)
C – Neoplasms	31,012 (0.8)	61,791 (0.3)
D – Disorders of Blood involving Immune System	30,791 (0.8)	123,797 (0.7)
E – Endocrine, Nutrition, and Metabolic Disorders	101,668 (2.5)	175,735 (1.0)
F – Mental and Behavioural Disorders	363,032 (9.0)	657,440 (3.7)
G – Diseases of Nervous System	103,126 (2.6)	285,510 (1.6)
H – Diseases of the Eye, Adnexa, Ear and Mastoid Process	33,316 (0.8)	593,309 (3.3)
I – Diseases of the Circulatory System	312,316 (7.7)	659,170 (3.7)
J – Diseases of the Respiratory System	327,755 (8.1)	1,481,326 (8.4)
K – Diseases of the Digestive System	213,696 (5.3)	1,172,130 (6.6)
L – Diseases of the Skin and Subcutaneous Tissue	45,659 (1.1)	796,004 (4.5)
M – Diseases of the Musculoskeletal System and Connective Tissue	186,194 (4.6)	1,443,302 (8.1)
N – Diseases of the Genitourinary System	190,734 (4.7)	1,131,151 (6.4)
O – Pregnancy, Childbirth, and the Puerperium	14,521 (0.4)	307,234 (1.7)
P – Certain conditions origination in the Perinatal Period	11 (0.0)	228 (0.0)
Q – Congenital Malformations, Deformations and Chromosomal Abnormalities	405 (0.0)	4178 (0.0)
R – Symptoms, Signs and Abnormal Clinical and Laboratory Findings	1,030,682 (25.6)	3,521,524 (19.9)
S,T – Injury, Poisoning and Certain Other Consequences of External Causes	833,644 (20.7)	3,704,644 (20.9)
U – External Coucals of Morbidity and Mortality	463 (0.0)	1602 (0.0)
Z – Factors Influencing Health Status and Contact with Health Services	79,206 (2.0)	1,019,098 (5.7)
Unreported	325 (0.0)	3484 (0.0)
**Emergency Department Setting**		
Urban	3,603,896 (89.4)	14,205,090 (80.1)
Rural	407,175 (10.1)	3,474,669 (19.6)
Unreported	20,472 (0.5)	53,338 (0.3)
**Visit outcome**		
Admitted to receiving facility from ED	1,411,377 (35.0)	1,337,110 (7.5)
Discharged from ED	2,620,166 (65.0)	16,395,987 (92.5)
**Returned to ED within 30 days of discharge**		
Yes	1,067,549 (26.5)	4,228,087 (23.8)
No	2,963,994 (73.5)	13,505,010 (76.2)

*Note*: *CTAS* Canadian Acuity and Triage Scale, *ED* emergency department

^a^Total of comorbidities present on ED arrival, included: hypertension, diabetes, chronic obstructive pulmonary disease, asthma, rheumatoid arthritis, congestive heart failure, bowel disease, cancer

^b^International Statistical Classification of Diseases and Related Health Problems 10th Revision

^c^Represents primary diagnosis of emergency department visit

**Fig. 2:**
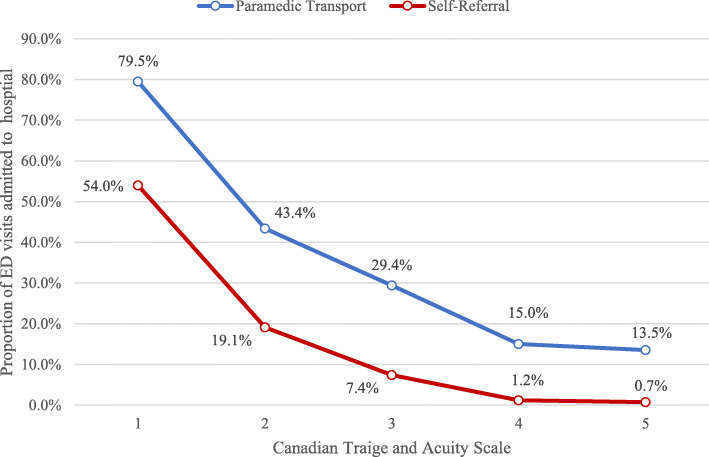
Ontario emergency department visits admitted to the receiving hospital, stratified by mode of transportation and acuity

Overall, 35% of patients transported by paramedics following a 9–1-1 emergency call were admitted to hospital compared with 7.5% of patients who self-referred to ED. Rates of hospital admission were higher when patients were transported by paramedics across all acuity scores. A line graph of admission rates for each CTAS acuity, by mode of transportation, is shown in Fig. 2.

Unadjusted and adjusted logistic regression models for hospital admission are shown in Table 2. Patients transported by paramedics were independently associated with hospital admission, after adjusting for clinically relevant factors (*OR* = 3.76, 95% *CI* = 3.74–3.77). Older age (40–64 years, *OR* = 1.57; 95% *CI* = 1.56–1.58; 65–105 years, *OR* = 3.29, 95% *CI* = 3.28–3.31), male sex (*OR* = 1.16, 95% *CI* = 1.16–1.17), emergent acuity (*OR* = 10.59, 95% *CI* = 10.52–10.66), urgent acuity (*OR* = 4.26, 95% *CI* = 4.23–4.29), more than two comorbidities, and attending an urban institution (*OR* = 1.13, 95% *CI* = 1.12–1.13), were also found to be significantly associated with hospital admission in our regression model.

**Table 2:** Unadjusted and adjusted associations between patient characteristics and hospital admission, emergency department patients, Ontario, Canada, April 1, 2015 to March 31, 2020

Characteristic	Unadjusted OR (95% CI)	Adjusted OR (95% CI)
I**ntercept**	–	0.02 (0.02–0.02)
**Mode of transportation,** paramedic transfer	6.61 (6.59–6.62)	3.76 (3.74–3.77)
**Sex,** male	1.15 (1.14–1.16)	1.16 (1.16–1.17)
**Age,** years		
18–39	–	–
40–64	1.89 (1.88–1.89)	1.57 (1.56–1.58)
65–105	5.82 (5.80–5.84)	3.29 (3.28–3.31)
**Acuity,** CTAS^a^		
Emergent	21.22 (21.01–21.34)	10.59 (10.52–10.66)
Urgent	7.17 (7.12–7.21)	4.26 (4.23–4.29)
Non-Urgent	–	–
**Comorbidities**^b^		
0–2	–	–
3–5	3.48 (3.47–3.49)	1.55 (1.54–1.55)
6–8	5.76 (5.67–5.86)	1.93 (1.89–1.97)
**Diagnostic Category,** ICD-10^c,d^		
Mental and Behavioural Disorders	–	–
Diseases of Circulatory System	2.32 (2.31–2.34)	1.21 (1.20–1.22)
Diseases of Respiratory System	0.74 (0.74–0.74)	0.73 (0.73–0.74)
Diseases of Digestive System	1.31 (1.30–1.32)	1.63 (1.62–1.64)
Diseases of the Skin and Subcutaneous Tissue	0.19 (0.19–1.20)	0.40 (0.40–0.40)
Diseases of the Musculoskeletal System and Connective Tissue	0.13 (0.13–0.13)	0.17 (0.17–0.18)
Diseases of Genitourinary System	0.49 (0.49–0.49)	0.55 (0.55–0.56)
Symptoms, Signs and Abnormal Clinical and Laboratory Findings	0.43 (0.42–0.43)	0.30 (0.30–0.31)
Injury, Poisoning and Certain Other Consequences of External Causes	0.23 (0.22–0.23)	0.27 (0.27–0.27)
Factors Influencing Health Status and Contact with Health Services	0.09 (0.09–0.09)	0.20 (0.20–0.20)
Other	0.63 (0.63–0.64)	0.71 (0.71–0.72)
**Geographic location,** urban	1.86 (1.85–1.86)	1.13 (1.12–1.13)
**Concordance statistic**^e^	–	0.86 (0.85–0.86)

*OR* Odds Ratio, *CI* Confidence Interval, *CTAS* Canadian Triage and Acuity Scale

^a^Emergent = CTAS 1, 2. Urgent = CATS 3. Non-Urgent = CTAS 4,5

^b^Total comorbidities present at ED visit, included: hypertension, diabetes, chronic obstructive pulmonary disease, asthma, rheumatoid arthritis, congestive heart failure, bowel disease, cancer

^c^International Statistical Classification of Diseases and Related Health Problems 10th Revision

^d^Represents primary diagnosis of emergency department visit

^e^Reported as Area Under the Receiver Operating Characteristic Curve (95% CI)

Two main diagnostic categorizations were associated with hospital admission, including diseases of the circulatory system (*OR* = 1.21, 95% *CI* = 1.20–1.22) and diseases of the digestive system (*OR* = 1.63, 95% *CI* = 1.62–1.64) with mental and behavioural disorders as the reference category. The area under the receiver operating characteristic curve (AUC) was 0.86, inferring this adjusted model is an excellent classifier to identify patient characteristics associated with hospital admission [25].

## Discussion

### Interpretation

Hospital admission subsequent to an ED visit was independently associated with paramedic transport after adjusting for important patient and visit characteristics. Characteristics of the male sex, older age groups, having a higher medical acuity, more than two comorbidities, seeking medical attention at an ED in an urban setting, and main diagnostic categories of the circulatory system and digestive system were also associated with admission in a population-based model. Of all patients admitted to Ontario hospitals from the ED, 51.4% were transported by paramedics compared against 48.6% for self-referral, despite self-referral composing much higher ED visit incidence in the population (81.5%). Paramedic transported patients had a higher probability of being admitted compared to self-referral (35.0% vs., 7.5%), and had higher probabilities of admission at each ordinal level of CTAS medical acuity.

The results of our study build on previous literature that analyzed the association between paramedic transport and hospital admission which were limited by small sample sizes and dated samples [8, 10, 15]. Our adjusted characteristics were consistent with the literature, finding older age groups and higher acuity are significant predictors of hospital admission [9, 11, 12]. The proportion of patients transported by paramedics that were admitted agrees with Canadian literature that studied the effects of paramedic utilization on hospital outcomes [26].

The underlying rationale for this relationship between paramedic transport and hospital admission is uncertain. A combination of clinical and non-clinical factors may compound with paramedic transport to influence disposition decision-making, when characteristics of patients transported are known to be higher in acuity and older compared with self-referred patients, as described in this study. Although an independent association between hospital admission and patients transported by paramedics was observed, other important outcomes such as frailty, formal support in the community (i.e., home care) and previous ED utilization were not studied. Further research is required to fully understand the impact of mode of transportation on hospital admission, and should incorporate additional potentially influential clinical and non-clinical characteristics to gain a better understanding of contextual factors influencing this relationship. Evidence of this association could inform and instigate future study concerning paramedic scope of practice and their associated implications on patient outcomes and hospital metrics. Research in paramedicine prioritizes study of emergent medical conditions and does not readily study patients with low medical acuities, an informative element of our analysis. Thus, our study contributes novel evidence to describe downstream implications of paramedic utilization on patient outcomes after hospital transfer of care, a result that is important to patients, clinicians and policymakers.

As paramedic utilization demands continue grow in Ontario, hospital admissions may also increase, which could further escalate hospital inpatient workloads and bed scarcity. The growing age cohort of complex older persons also increases the incidence of delayed-discharge, commonly referred to as alternate level of care (ALC) in Canada, further impeding hospital throughput [27]. Identification and correction to barriers in outpatient care access may help to improve the wellbeing of patients at risk of unwarranted paramedic utilization. Additionally, alternative care pathways for paramedic transport to sub-acute care settings may assist in mitigating hospital congestion, diverting patient cases that have low risk of medical debility, given evidence-based protocols could be established. The results of this study are likely to be generalizable to other provinces and territories of Canada, as paramedic service delivery models and hospitals are largely constructed using the similar organizational framework and universal healthcare payment coverages.

### Limitations

Due to the nature of regression analysis in cohort studies, a causal relationship between mode of transportation to ED and hospital admission could not be determined. Other patient and visit characteristics may influence the results that were not included in this study; however, they were not accessible through the NACRS administrative database. Incorporation of comorbidities in this study were limited to those readily collected in administrative data by IC/ES. Primary diagnoses could only be grouped into broadly assigned categories based on their header in ICD-10.

## Conclusion

Paramedic transport to the ED was associated hospital admission in a population-based cohort of adult patients who received triage in an Ontario ED. Patients transported by paramedics had higher rates of admission across all CTAS acuities compared to self-referral. We demonstrated that the mode of transportation may have an influence on ED disposition decision-making, and support future research to target some patients that may be able to be transported to alternative sub-acute care settings as a means to decrease hospital admission rates.

## Supplementary Information

**Additional file 1.**

## Data Availability

All aggregate data herein are accessible to other interested parties by application to the corresponding author.

## Abbreviations

CTAS: Canadian Triage and Acuity Scale
ED: Emergency department
IC/ES: Institute for Clinical Evaluative Sciences
NACRS: National Ambulatory Care Reporting System
OR: Odds ratio

## References

[1] Canadian Institute for Health Information. Inpatient Hospitalization, Surgery and Newborn Statistics, 2018–2019. Ottawa: CIHI; 2020.

[2] Forster AJ, Stiell I, Wells G, Lee AJ, van Walraven C (2003). The effect of hospital occupancy on emergency department length of stay and patient disposition. Acad Emerg Med Off J Soc Acad Emerg Med.

[3] Kone AP, Mondor L, Maxwell C, Kabir US, Rosella LC, Wodchis WP (2021). Rising burden of multimorbidity and related socio-demographic factors: a repeated cross-sectional study of Ontarians. Can J Public Health.

[4] Devlin R. Hallway health care: a system under strain. Toronto: Government of Ontario; 2019. Available from: https://www.health.gov.on.ca/en/public/publications/premiers_council/docs/premiers_council_report.pdf.

[5] OECD (2020). Hospital beds (indicator).

[6] Government of Canada SC. Population estimates, quarterly. 2021 [cited 2021 Jun 24]. Available from: https://www150.statcan.gc.ca/t1/tbl1/en/tv.action?pid=1710000901

[7] Gabayan GZ, Sarkisian CA, Liang L-J, Sun BC (2015). Predictors of admission after emergency department discharge in older adults. J Am Geriatr Soc.

[8] Lin D, Worster A (2013). Predictors of admission to hospital of patients triaged as nonurgent using the Canadian Triage and Acuity Scale. CJEM.

[9] Lo AX, Flood KL, Biese K, Platts-Mills TF, Donnelly JP, Carpenter CR (2016). Factors Associated With Hospital Admission for Older Adults Receiving Care in U.S. Emergency Departments. J Gerontol A Biol Sci Med Sci.

[10] Lucke JA, de Gelder J, Clarijs F, Heringhaus C, de Craen AJM, Fogteloo AJ (2018). Early prediction of hospital admission for emergency department patients: a comparison between patients younger or older than 70 years. Emerg Med J.

[11] Marcusson J, Nord M, Dong H-J, Lyth J (2020). Clinically useful prediction of hospital admissions in an older population. BMC Geriatr.

[12] Morris JN, Howard EP, Steel K, Schreiber R, Fries BE, Lipsitz LA (2014). Predicting risk of hospital and emergency department use for home care elderly persons through a secondary analysis of cross-national data. BMC Health Serv Res.

[13] Lewis Hunter AE, Spatz ES, Bernstein SL, Rosenthal MS (2016). Factors Influencing Hospital Admission of Non-critically Ill Patients Presenting to the Emergency Department: a Cross-sectional Study. J Gen Intern Med.

[14] Hajjaj F, Salek M, Basra M, Finlay A (2010). Non-clinical influences on clinical decision-making: a major challenge to evidence-based practice. J R Soc Med.

[15] Parker CA, Liu N, Wu SX, Shen Y, SSW L, MEH O (2019). Predicting hospital admission at the emergency department triage: A novel prediction model. Am J Emerg Med.

[16] Sun Y, Heng BH, Tay SY, Seow E (2011). Predicting Hospital Admissions at Emergency Department Triage Using Routine Administrative Data. Acad Emerg Med.

[17] Government of Ontario M of H and L-TC. Emergency Health Services Land Ambulance Program - Emergency Health Services - Programs and Services - Health Care Professionals - MOHLTC [Internet]. Government of Ontario, Ministry of Health and Long-Term Care; Available from: http://www.health.gov.on.ca/en/pro/programs/emergency_health/land/responsetime.aspx

[18] Strum RP, Tavares W, Worster A, Griffith LE, Rahim A, Costa AP (2021). Development of the PriCARE classification for potentially preventable emergency department visits by ambulance: a RAND/UCLA modified Delphi study protocol. BMJ Open.

[19] von Elm E, Altman DG, Egger M, Pocock SJ, Gøtzsche PC, Vandenbroucke JP (2007). Strengthening the reporting of observational studies in epidemiology (STROBE) statement: guidelines for reporting observational studies. BMJ.

[20] Lane DJ, Blanchard IE, Buick JE, Shaw M, AD MR (2021). Changes in presentation, presenting severity and disposition among patients accessing emergency services during the first months of the COVID-19 pandemic in Calgary, Alberta: a descriptive study. Can Med Assoc Open Access J.

[21] Canadian Institute for Health Information. NACRS Data Elements, 2021–2022. Ottawa: CIHI; 2021. https://www.cihi.ca/sites/default/files/document/nacrs-data-elements-2021-2022-en.pdf.

[22] Li G, Lau JT, ML MC, Schull MJ, Vermeulen M, Kelen GD (2007). Emergency Department Utilization in the United States and Ontario, Canada. Acad Emerg Med.

[23] Bullard MJ, Unger B, Spence J, Grafstein E, the CTAS National Working Group (2008). Revisions to the Canadian Emergency Department Triage and Acuity Scale (CTAS) adult guidelines. CJEM.

[24] Mowbray FI, Aryal K, Mercier E, Heckman G, Costa AP (2020). Older emergency department patients: does baseline care status matter?. Can Geriatr J CGJ.

[25] Hosmer DW Jr, Lemeshow S, Sturdivant RX. Applied Logistic Regression. New Jersey: Wiley; 2013. p. 528.

[26] Tavares W, Drennan I, Van Diepen K, Abanil M, Kedzierski N, Spearen C (2017). Building capacity in healthcare by re-examining clinical Services in Paramedicine. Prehospital Emerg Care.

[27] Costa AP, Hirdes JP. Clinical Characteristics and Service Needs of Alternate-Level-of-Care Patients Waiting for Long-Term Care in Ontario Hospitals. Healthc Policy. 2010;6(1) [cited 2021 Jul 8]Available from: https://www.longwoods.com/content/21899/healthcare-policy/clinical-characteristics-and-service-needs-of-alternate-level-of-care-patients-waiting-for-long-term. PMC2929891 21804837

[28] Government of Ontario (2014). Personal Health Information Protection Act, 2004, S.O. 2004, c. 3, Sched. A.

[29] Government of Ontario (2021). Ontario Regulation 329/04: General, Personal Health Information Protection Act.

